# Drought increases the frequencies of fungal functional genes related to carbon and nitrogen acquisition

**DOI:** 10.1371/journal.pone.0206441

**Published:** 2018-11-21

**Authors:** Kathleen K. Treseder, Renaud Berlemont, Steven D. Allison, Adam C. Martiny

**Affiliations:** 1 Department of Ecology and Evolutionary Biology, University of California Irvine, Irvine, California, United States of America; 2 Department of Biological Sciences, California State University Long Beach, Long Beach, California, United States of America; 3 Department of Earth System Science, University of California Irvine, Irvine, California, United States of America; North Carolina State University, UNITED STATES

## Abstract

Although water is a critical resource for organisms, microbially-mediated processes such as decomposition and nitrogen (N) transformations can endure within ecosystems even when water is scarce. To identify underlying mechanisms, we examined the genetic potential for fungi to contribute to specific aspects of carbon (C) and N cycling in a drought manipulation in Southern California grassland. In particular, we measured the frequency of fungal functional genes encoding enzymes that break down cellulose and chitin, and take up ammonium and amino acids, in decomposing litter. Furthermore, we used “microbial cages” to reciprocally transplant litter and microbes between control and drought plots. This approach allowed us to distinguish direct effects of drought in the plot environment versus indirect effects via shifts in the microbial community or changes in litter chemistry. For every fungal functional gene we examined, the frequency of that gene within the microbial community increased significantly in drought plots compared to control plots. In contrast, when plot environment was held constant, frequencies of these fungal functional genes did not differ significantly between control-derived microbes versus drought-derived microbes, or between control-derived litter versus drought-derived litter. It appears that drought directly selects for fungi with the genetic capacity to acquire these specific C- and N-containing compounds. This genetic trait may allow fungi to take advantage of ephemeral water supplies. Altogether, proliferation of fungi with the genetic capacity for C and N acquisition may contribute to the maintenance of biogeochemical cycling under drought.

## Introduction

Fungi can be remarkably drought tolerant—they can remain active and even grow under extremely dry conditions [1, 2]. Similarly, fungal abundance can increase under drought [3–8]. Indeed, fungi have a number of traits that can mitigate drought stress, including osmolytes, thick cell walls, and melanin [9–11]. In addition, because filamentous fungi produce hyphae that can extend up to meters, they can also forage for water across the soil matrix [12–15]. Altogether, fungi can be more drought-tolerant than bacteria [1, 3, 9].

Fungi are also important players in ecosystems [16]. They conduct nitrogen (N) transformations such as N mineralization and microbial N immobilization [17–20]. Moreover, fungi can degrade particularly recalcitrant carbon (C) compounds, including lignin, lignocellulose, and crystalline cellulose [21–24]. Thus, drought resistance by fungi might enable N transformations and decomposition to continue under dry conditions.

In fact, a number of field observations have indicated that decomposition and N transformations do not necessarily decline under drought conditions [10, 25–27]. Often, wood decomposition slows but remains measurable under dry conditions [28–32]. Soil respiration can continue even during dry seasons when plants are dormant, especially in arid and semi-arid ecosystems [33]. Likewise, a recent meta-analysis indicated that N mineralization does not generally change with drought [34]. In fact, N mineralization can even increase with drought in some ecosystems [25, 26, 34–36]. Nevertheless, it is not clear to what extent fungi contribute to drought resistance of these processes. We might expect fungi to dominate these processes under drought conditions, given their general tolerance to drought [1].

We can examine this issue by measuring the prevalence of relevant fungal functional genes [37]. One advantage of this approach is that we can use DNA sequences to link genes to taxa [38]. This way, we can focus on the potential contributions of fungi to C and N cycling under natural conditions. The annotation of fungal functional genes is still in its early stages [38, 39]. Even so, certain functional genes that target C and N have been characterized in fungi or, more broadly, eukaryotes [11]. These include genes encoding extracellular chitinases, which break down chitin; amino acid permeases and ammonium transporters, which take up N from the environment; and cellulose-targeting AA9 enzymes, which can degrade crystalline cellulose [40–45].

**Fig 1:**
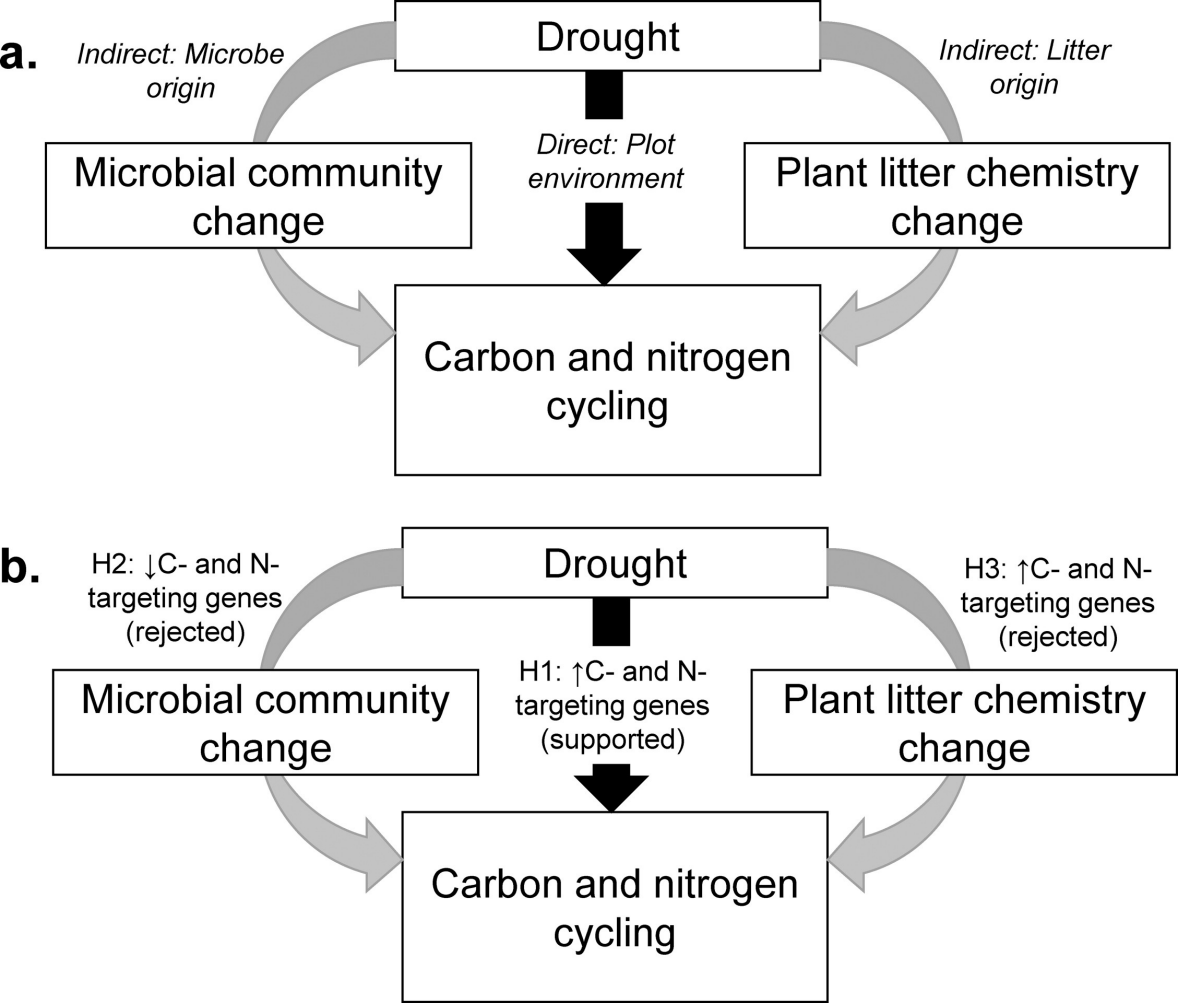
Conceptual framework of drought effects on fungal functional genes related to acquisition of carbon (e.g., cellulose-targeting AA9 genes) and nitrogen cycling (e.g., extracellular chitinase, amino acid permease, and ammonium transporter genes). Drought can directly alter gene profiles via changes in the plot environment, or indirectly shift them via changes in the microbial community or plant litter chemistry (a). We tested predictions from three hypotheses, each related to a specific effect (b). Only the direct effect of drought in the plot environment altered fungal functional genes as predicted.

Fundamentally, fungi require water to conduct decomposition and transform nitrogen [46]. Extracellular enzymes reach their targets by diffusing through water; in turn, fungi take up small C and N compounds via diffusion [47]. When soils or litter dry, water becomes restricted to small pockets or films within the soil [26, 48]. This hydrological discontinuity can impede diffusion of enzymes and nutrients [49]. Fungi can offset this constraint over the short term by producing more C- and N-targeting enzymes, to better take advantage of scarce or ephemeral water pockets [49]. Thus, we hypothesized that drought should *directly* increase the frequencies of C- and N-targeting enzymes in the microbial community, because fungi with greater genetic capacity for production of C- and N-targeting enzymes will have a competitive advantage (Hypothesis 1, Fig 1).

In addition, drought could *indirectly* influence the distribution of fungal functional genes targeting C and N by shifting the composition of the microbial community (sensu [10]). Frequently, water availability can significantly affect fungal community composition, across climatic zones as well as in manipulations (e.g., [50–54]). Moreover, closely-related fungi tend to respond to drought more similarly than do less-related fungi [9, 55]. Because closely-related fungi tend to share traits that influence C- and N-cycling as well [11, 56], it is possible that drought could select for fungal taxa that are associated with particular C- and N-targeting genes. Specifically, we expect that evolutionary trade-offs may select for species with limited genetic capacity for production of C- and N-targeting enzymes, because resources invested in drought tolerance (e.g., osmolytes or thicker cell walls) will not be available for production of the enzymes [11, 49]. These evolutionary trade-offs may play out over relatively long time scales. Accordingly, we hypothesized that drought-induced microbial community shifts will indirectly reduce the prevalence of fungal C- and N-targeting genes over the long term (Hypothesis 2, Fig 1).

Drought can also *indirectly* change fungal functional genes by altering the chemical composition of plant litter that the fungi are decomposing (sensu [57, 58]). For example, in a Southern Californian grassland, Allison et al. [3] reported that leaf litter grown under drought conditions had higher C:N ratios and lower concentrations of cellulose and hemicellulose. In other words, N and cellulose were less available to fungi in drought-derived litter. Accordingly, for this ecosystem, we hypothesized that drought-derived litter would be associated with greater frequencies of cellulose- and N-targeting genes, because fungi that invest in acquisition of limiting resources should be favored (Hypothesis 3, Fig 1).

**Fig 2:**
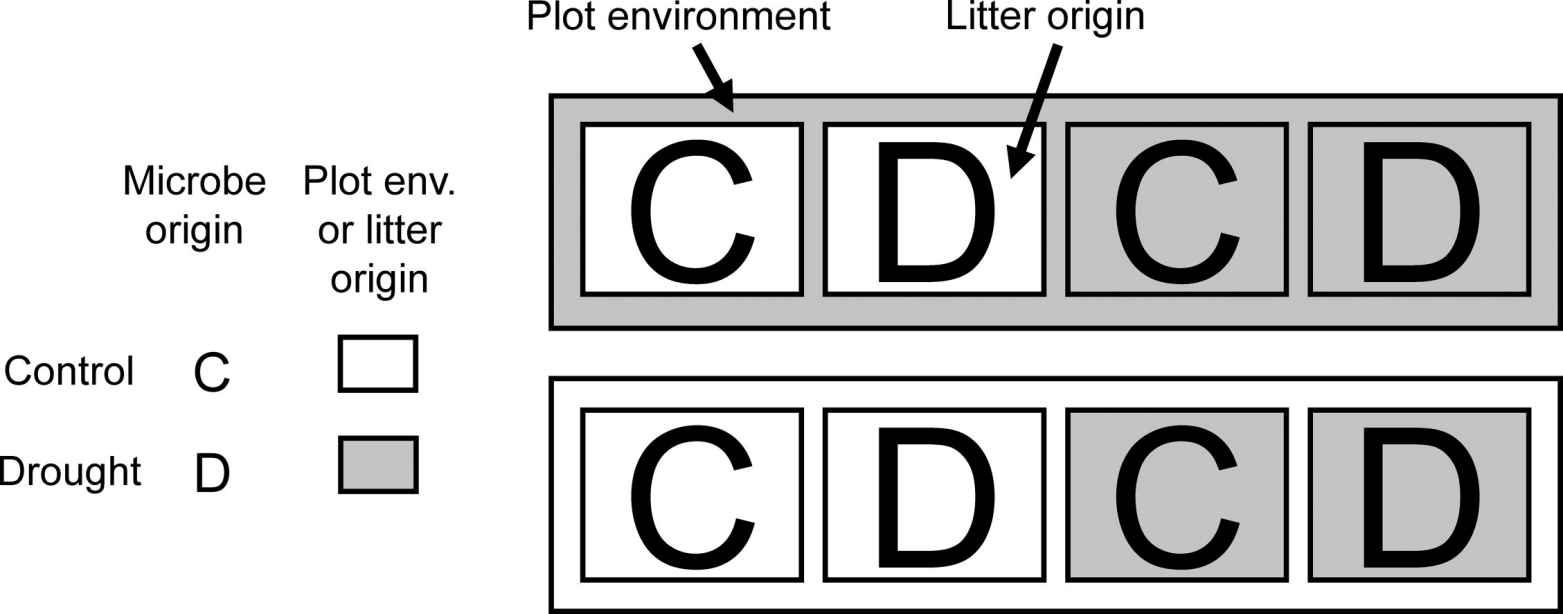
Reciprocal transplant design for decomposition experiment. **We crossed plot environment, microbe origin, and litter origin in a fully factorial design.** There were two levels for each treatment: control and drought. Control plots were unmanipulated, while drought plots received ~50% less rainfall.

To test our hypotheses, we used “microbial cages” in a reciprocal litter transplant to independently manipulate direct effects via drought in the environment versus indirect effects via shifts in microbial community composition, or indirect effects via changes in litter chemistry (Fig 2, ref. [3]). This field experiment was located in a Southern California grassland subjected to long-term drought. Following decomposition, we shotgun-sequenced DNA in each litterbag [54], and then measured the frequency of fungal genes involved in N acquisition (extracellular chitinases, amino acid permeases, and ammonium transporters) and C acquisition (enzymes that are associated with cellulose breakdown). Previous work in this experiment has established that drought alters fungal community composition [54, 55, 59].

## Materials and methods

### Field site

We examined this issue in a drought experiment located in a grassland in coastal Orange County, California USA (33° 44’ N, 117° 42’ W, 365 m elevation). Orange County Parks and the Irvine Ranch Conservancy provided field permits and granted access to this field site. Allison et al. [3] describe this field manipulation in detail. Briefly, the grassland is dominated by the native perennial grass *Nassella pulchra*, as well as exotic grasses and annual forbs [60]. Mean annual rainfall is 325 mm y^-1^, and it occurs primarily between October and April. Mean annual temperature is 17°C.

The drought experiment commenced in February 2007. Plots were arranged in eight blocks. In each block, one plot experienced reduced rainfall, and the other remained unmanipulated as a control. Each plot was 3.3 x 9.3 m. We reduced rainfall each winter by covering the plots with clear polyethylene during a subset of rainstorms to reduce annual precipitation by 50%. This treatment reduced rainfall from 369 to 194 mm during the 2009–2010 winter, and from 540 to 213 mm during the 2010–2011 winter.

### Microbial cages

We followed similar protocols to those described in Treseder et al. [61]. First, we collected plant litter to decompose. We collected litter from four 0.07 m^2^ quadrats haphazardly located in each drought plot and control plot on 29 June, 2 July, and 14 September 2010. (We used multiple collection dates, because deep-rooted annual forbs senesced later in the season than did the other plants.) We pooled and hand-homogenized litter from all plots within each treatment.

Second, we assembled “microbial cages” within which we decomposed the plant litter (sensu [62]). Microbial cages are litterbags made from nylon membrane with 0.45 μm pores. Fungi and most larger bacteria cannot pass through these pores, but water, nutrients, and unusually small bacteria can. We added 2 g air-dried litter to each cage, and then sterilized the completed cages with at least 22 kGy of gamma irradiation. We verified sterility by placing subsamples of irradiated litter in 50 mL sterile tubes with either potato dextrose broth (for fungi growth; Becton, Dickinson and Company; Franklin Lakes, NJ) or lysogeny broth (for bacterial growth, Fisher Scientific, Pittsburgh PA), shaking at 37°C (three days for fungi and 12 hours for bacteria). Then, we plated out 100 μl of the media on potato dextrose plates or lysogeny broth. We included a positive control (i.e., non-irradiated litter) to confirm that this procedure yielded colony growth.

Third, microbial inoculum was collected from control and drought plots to add to the microbial cages. On 30 November 2010, we hand-collected three haphazardly located litter samples (~5 g each) from each of the eight control and eight drought plots. We generated two batches of microbial inoculum: “control microbes” and “drought microbes”, by combining litter samples within each treatment. The inoculum was air dried, ground in a Wiley mill (1 mm mesh), and then added as 50 mg aliquots to microbial cages containing sterilized litter.

### Reciprocally transplanted litter

Prior to decomposition, litter from the drought plots had significantly higher C:N ratios, concentrations of total C (i.e., %C), lignin, sugars, starch, fat, and protein, compared to litter from control plots [3]. In contrast, cellulose and hemicellulose concentrations were significantly lower in the drought litter than the control litter [3]. Nitrogen concentrations (i.e, %N) did not differ significantly by litter origin [3]. Litter chemistry was originally analyzed by Allison et al. [3] from the same litter used in the current study. They determined total C, total N, and C:N by elemental analysis, and the other fractions by near infrared spectroscopy.

For the current study, we isolated the effects of these changes in litter chemistry by decomposing control-derived (“control litter”) and drought-derived plant litter (“drought litter”) in the control and drought plots (Fig 2). Accordingly, we placed four microbial cages (one of each microbe x litter combination) in each of eight control plots and eight drought plots. The cages were placed within standing grass litter. One corner of each microbial cage was tethered to the soil surface, and the remainder rested on the standing grass litter. This way, the orientation of the microbial cage matched that of the standing litter. Each cage contained either control or drought litter inoculated with control or drought microbes in a factorial design. Thus, there were 64 microbial cages total. Microbial cages were incubated in the plots for three months, from 15 December 2010 to 3 March 2011.

### DNA sequencing and annotation

For sequencing and annotation, we followed procedures previously described in Berlemont et al [8]. First, to balance cost limitations of sequencing with replication, we pooled eight plots from each treatment (each plot sampled and extracted separately) into two duplicate samples for sequencing. Then, we ground about 0.05 g of each litter sample in a mixer, subjected it to direct DNA isolation as described before [63], and normalized for the amount of leaf litter material used in the extraction. We used Covaris to fragment DNA to 300 bp. We then pooled equal amounts of DNA extracts into two replicates for sequencing. From the litter manipulation, 64 microbial cages were processed, yielding 16 metagenomic libraries. We prepared metagenomic libraries by using a Truseq library kit (Illumina, San Diego, CA, USA), and then sequenced them via Illumina HiSeq2000 (100 bp-paired ends). We treated sequences as single reads for subsequent analysis. We uploaded sequences onto the MG-RAST server [64] for overall taxonomic annotation, and to make them publically accessible [54]. Altogether, we obtained 111,110,681 reads totaling 23.2 Gbp after quality control.

### Fungal functional genes

We identified the frequencies of fungal functional genes in sequenced metagenomes by performing a BLAST search [65, 66] using a custom reference database derived from the InterPro database (https://www.ebi.ac.uk/interpro/) using the following steps. First, we identified functional genes of interest. We focused on functional genes in the database that (1) influenced C and N cycling in ecosystems, (2) had been experimentally characterized in fungi, and (3) had been sufficiently sequenced to distinguish eukaryote- versus bacterial-derived genes [following 11]. Only a subset of functional genes had been annotated well enough to meet these criteria [38]. N-targeting genes included extracellular chitinases, which break down chitin (e.g., GH18 family, IPR001223) [40, 41]; ammonium transporter genes, which take up ammonium from soil (e.g., AMT2, IPR001905) [42–44]; and amino acid permease genes, which take up amino acids from soil (e.g., AAP1 and GAP1, IPR004762). For C-targeting genes, we focused on lytic polysaccharide monooxygenase family AA9 (IPR005103) [45, 67]. Extracellular enzymes of this family contribute to the breakdown of relatively recalcitrant forms of cellulose, including highly crystalline cellulose as well as cellulose molecules that are cross-linked with lignin [22, 68, 69]. Finally, for each function of interest, taxonomically resolved sequence datasets were retrieved from the InterPro database and combined into a single reference sequence database. More precisely, for cellulose-targeting AA9 genes and chitinase genes, sequences in the reference database were assigned to the phylum Ascomycota or Basidiomycota. We examined genes from these two phyla separately, because ascomycetes and basidiomycetes can differ in traits related to drought responses or ecosystem dynamics [11]. For ammonium transporter genes, we restricted our analyses to genes characterized in the phylum Ascomycota, as this gene family has not yet been well annotated in the Basidiomycota. For amino acid permease genes, kingdom-level annotations were not available. Instead, we focused on amino acid permease genes that could be assigned to Eukaryota. When performing the BLAST, positive matches were considered for sequences with e-value ≤10^−5^, to account for the size of the sequence database [66, 70]. Using this cut-off, 0.09% of the microbial cage sequences were identified as fungal functional genes of interest.

### Fungal hyphal length

To compare changes in fungal functional genes with changes in fungal abundance under drought, we measured fungal hyphal length in the same litter samples. These fungal hyphal lengths were originally reported by Allison et al. [3], who determined them using a modified procedure of Sylvia [71]. Litter subsamples (0.1 g) were ground to 1–2 mm, dried at 60°C, and dispersed in 10 ml sodium hexametaphosphate solution (0.395% mass/volume) with vigorous stirring. A 1.5 ml subsample of this solution was vacuum-filtered through a 0.2-μm nylon filter (Millipore, Billerica, Massachusetts, USA) and stained with acid fuchsin. This process was repeated with a second 1.5 ml subsample, and both filters were dried and mounted on a microscope slide with Permount (Fisher Scientific, Pittsburgh, Pennsylvania, USA). After drying at 20°C overnight, hyphal lengths (m/g dry litter) were determined with a Nikon Eclipse E400 microscope (Nikon Instruments, Melville, New York, USA) in phase contrast mode under 100X magnification using the grid-intercept method [72] and 50 grids per filter.

### Statistics

To test our hypotheses, we conducted a series of fully-factorial analyses of variance (ANOVAs). In each case, plot environment, microbe origin, and litter origin were the independent variables. The dependent variable was frequency of the functional gene of interest (e.g., amino acid permease, cellulose-targeting AA9 basidiomycete genes, etc) as number of hits per 100,000 reads (including DNA from fungi, bacteria, etc.). Where appropriate, we used Tukey post hoc comparisons to check for pairwise differences among treatments. Data distributions met the assumptions of ANOVA, so we did not transform them. If drought in the plot environment were associated with increases in frequencies of C- and N-targeting genes, Hypothesis 1 would be supported. Conversely, significant decreases in frequencies of C- and N-targeting genes in drought microbes versus control microbes would support Hypothesis 2. Likewise, decreases in these genes in drought litter compared to control litter would support Hypothesis 3.

## Results

### Functional genes

#### Direct effect: Plot environment

**Fig 3:**
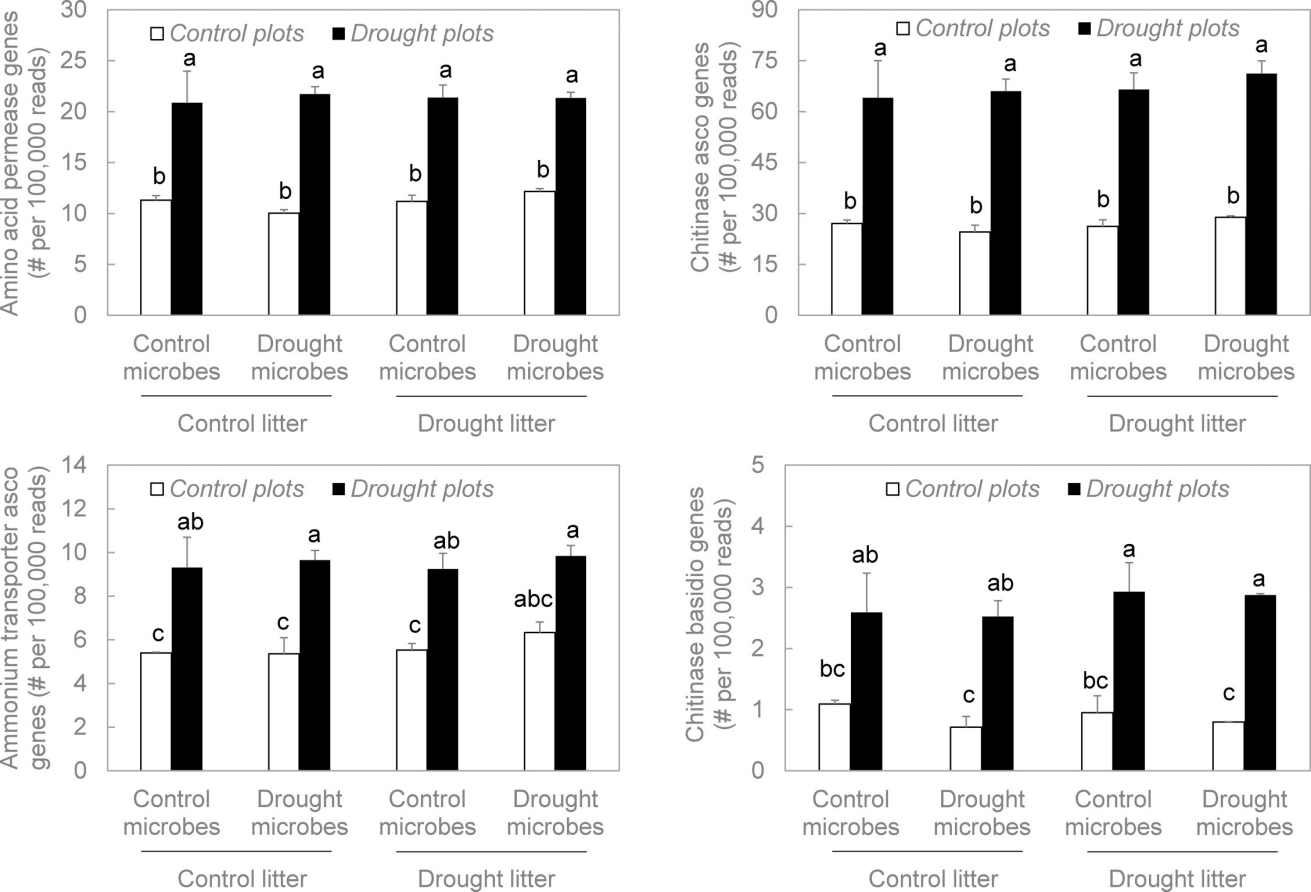
Frequencies of fungal functional genes related to N acquisition in the reciprocal litter transplant. For each gene family, plot environment significantly altered gene frequencies (P < 0.001 in all cases, S1 Table). Microbial origin and litter origin were not significant in any case. Treatment combinations associated with different letters were significantly different from one another. Bars are means +1SE of 2 replicates. Asco = ascomycete, basidio = basidiomycete.

**Fig 4:**
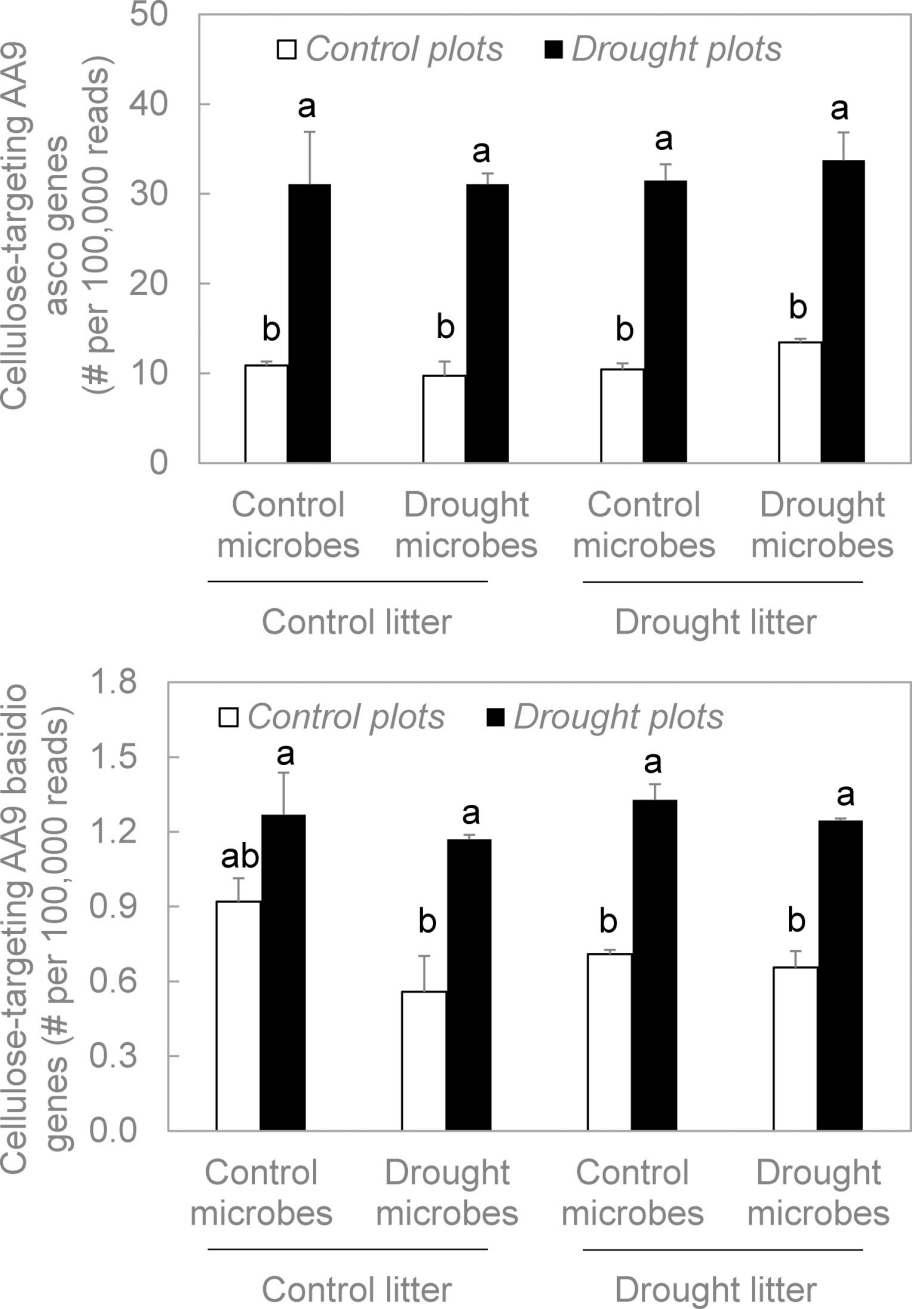
Frequencies of fungal functional genes related to C acquisition in the reciprocal litter transplant. Cellulose-targeting AA9 is an enzyme that targets crystalline cellulose. For each gene family, plot environment significantly altered gene frequencies (S1 Table, P < 0.001 in both cases). Microbial origin and litter origin were not significant in any case. Treatment combinations associated with different letters were significantly different from one another. Bars are means +1SE of 2 replicates. Asco = ascomycete, basidio = basidiomycete.

We had hypothesized that drought in the plot environment would directly induce an increase in frequencies of fungal functional genes related to C- and N-acquisition. Indeed, this hypothesis was significantly and strongly supported for all functional genes we examined: ammonium transporter genes, amino acid permease genes, chitinase genes, and cellulose-targeting AA9 genes (P < 0.001 in all cases, Figs 3 and 4, S1 Table). Moreover, this response was consistent across taxonomic groups; ascomycetes and basidiomycetes each displayed significant increases in chitinase (Fig 3) and cellulose-targeting AA9 gene frequencies (Fig 4). Accordingly, these results supported our first hypothesis.

#### Indirect effect: Microbial origin

Conversely, we rejected our second hypothesis, which stated that microbes from the drought treatment should contain lower frequencies of C- and N-targeting fungal genes than those from the control treatment, when plot environment and litter origin were held constant. For every functional gene and taxonomic group we assessed, there were no significant differences in gene frequency between drought-derived microbes and control-derived microbes (P > 0.05 in all cases, Figs 3 and 4, S1 Table). In addition, microbial origin did not significantly interact with plot environment or litter origin to influence gene frequencies.

#### Indirect effect: Litter origin

Moreover, we rejected our third hypothesis, which proposed that litter origin should indirectly affect fungal functional genes by augmenting genes related to C- and N-acquisition. In no case did frequencies of these genes change significantly for drought- versus control-derived litter (P > 0.05 in all cases, Figs 3 and 4, S1 Table). Likewise, no interactions between litter origin, plot environment, or microbial origin were significant.

### Fungal hyphal length

**Fig 5:**
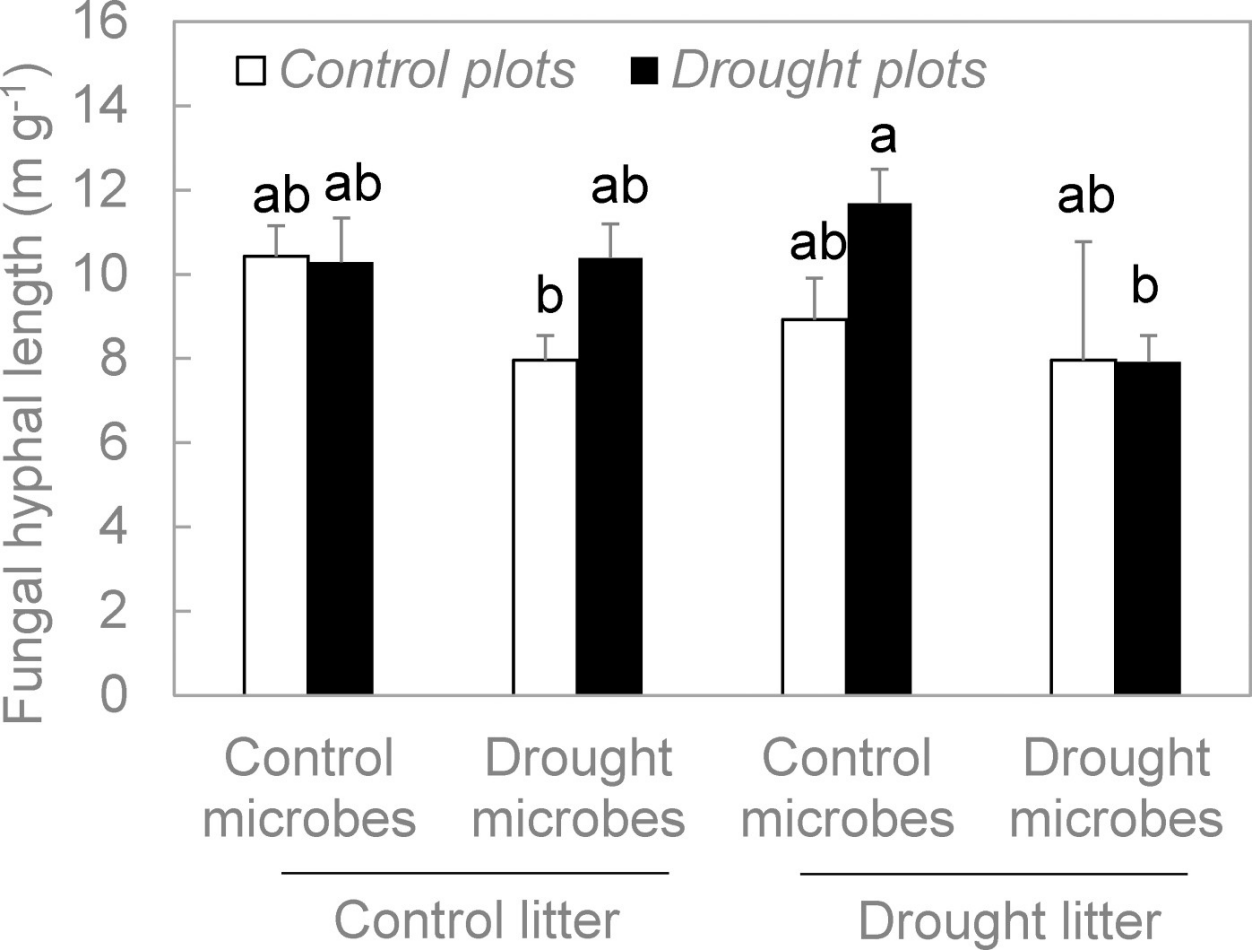
Fungal hyphal lengths (an index of abundance) in litter from a reciprocal transplant. Hyphal lengths were significantly greater in drought versus control plots (P = 0.026), and significantly lower in drought-derived versus control-derived microbial communities (P = 0.002). In addition, there was a significant plot environment x microbe origin x litter origin interaction (P = 0.018, S1 Table). Treatment combinations associated with different letters were significantly different from one another. Bars are means +1SE of 2 replicates.

As with fungal functional genes, plot environment was associated with a significant change in hyphal lengths. Specifically, hyphal lengths were greater in the drought plots than the control plots (P = 0.026, Fig 5, S1 Table). Nevertheless, treatment effects on fungal hyphal lengths departed from those of the functional genes in two respects. First, microbe origin significantly affected hyphal lengths, with lower fungal abundance in the drought-derived community than in the control-derived community (P = 0.002). Second, microbe origin and litter origin mediated the plot environment effect, which yielded a significant three way interaction between these factors (P = 0.018). Specifically, drought in the plot environment tended to increase fungal hyphal length just for drought microbes growing on control litter, and control microbes growing on drought litter (Fig 5). All other effects and interactions were non-significant (P > 0.05).

## Discussion

We used metagenomic sequencing of the fungal community in decomposing litter to assess how the prevalence of C- and N-targeting functional genes responded to drought (Fig 1). Specifically, we examined functional genes encoding cellulose-targeting AA9 enzymes (for C); and extracellular chitinases, amino acid permeases, and ammonium transporters (for N). In support of our first hypothesis, direct, short-term effects of drought in the plot environment markedly increased frequencies of every C- and N-targeting fungal functional gene we examined (Figs 3 and 4). In contrast, indirect effects via microbial community shifts or changes in litter chemistry were consistently non-significant. Thus, we rejected our second and third hypotheses that both of these indirect effects would decrease the frequencies of C- and N-targeting genes. Taken together, we may expect fungal contributions to these specific elements of C- and N-cycling to increase under drought, at least in the short-term.

Fungal abundance also increased significantly in response to drought (Fig 5). However, this change was more subtle than that of the functional genes. In addition, other significant treatment effects on fungal abundance were not mirrored in functional gene frequency. For example, microbes from the drought treatment yielded significantly lower fungal abundance than did those from the control treatment, when all were grown under common conditions. This effect of microbial origin was not evident for any of the functional genes (Figs 3 and 4). Although the increase in fungal abundance in the drought environment may have contributed somewhat to the increase in functional gene frequencies, other mechanisms were likely involved. Next, we examine these potential mechanisms.

### Direct effect: Plot environment

Drought in the plot environment consistently increased the frequencies of the C- and N-targeting fungal genes we examined (Figs 3 and 4). Given that the litter decomposed in the plot treatments for only three months, this effect appeared relatively quickly. One possible explanation is that drought selected for fungi with greater genetic capacities to produce C- and N-targeting enzymes, because these individuals could better take advantage of water when it was available. For instance, in a theoretical analysis, Manzoni et al. [49] suggested that microbes with high capacity for extracellular enzyme production could construct the enzymes quickly after a rainfall, improving their ability to compete for compounds that had accumulated in the environment during the previous dry spell. Possession of these functional genes may have conferred competitive advantages. Martiny et al. [54] noted that drought in the plot environment altered fungal community composition in the same litter samples we examined for the current study. In addition, fungi generally appeared more drought-tolerant than bacteria in this study. In particular, bacterial abundance declined significantly with drought, while fungal abundance increased significantly (Fig 5, ref. [3]). This shift between fungi and bacteria could have also contributed to higher frequencies of these fungal functional genes within the microbial community.

Of course, the presence of a particular gene in the fungal community does not necessarily indicate that the gene is expressed. Nevertheless, certain C- and N-dynamics in the decomposing litter are consistent with the shift in fungal functional genes we observed. Specifically, Alster et al. [73] reported that drought in the plot environment increases potential activities of the extracellular enzymes β-glucosidase and cellobiohydrolase, which break down cellulose, as well as N-acetyl-β-D-glucosaminidase, which degrades chitin and is often associated with fungal activity. Likewise, cellulose concentrations in decomposed litter are lower in the drought plot environment than in the control plot environment, indicating that cellulose is broken down faster under drought [3]. However, litter N concentrations are higher after decomposition in the drought plots [3]. This response is not consistent with the increase in frequencies of N-targeting fungal genes we observed in that plot environment, because the increase in genes—if expressed—should have removed N polymers from the decomposing litter. Bacterial functional genes also shifted in the drought versus control plot environment [54], which may have contributed to the decrease in N loss from the litter decomposed under drought.

### Indirect effect: Microbe origin

With respect to the microbe origin treatment, we rejected our hypothesis that over a longer time scale, evolutionary trade-offs should select against fungal genes targeting C and N acquisition in microbial communities exposed to drought. In fact, frequencies of these functional genes did not differ significantly between drought- and control-derived microbial communities, when litter origin and plot environment were held constant (Figs 3 and 4). Thus, we found little evidence for the evolutionary trade-offs proposed by Treseder and Lennon [11].

Remarkably, even though frequencies of fungal functional genes targeting C and N did not change significantly with microbial origin, fungal community composition and abundance did. The drought treatment had been imposed for 3.75 years before we collected microbial inoculum for the decomposition experiment. By that time, fungal community composition had shifted, with several ascomycete taxa (e.g., Davidiellaceae, Dothioraceae, and Didymosphaeriaceae) declining and certain basidiomycete taxa (e.g., Tremellaceae and Atheliaceae) increasing [54, 55]. Even after the fungi had been transplanted and grown in similar environments for the three-month duration of our decomposition experiment, the original differences in fungal community composition remained [54]. In addition, fungi were less abundant in the drought-derived versus the control-derived microbial communities (Fig 5). Likewise, Alster et al. [73] reported that microbial origin effects on potential activities of extracellular enzymes were subtle; the only significant effect was a minor increase in activity of the chitinase N-acetyl-β-D-glucosaminidase in the drought-derived microbes. Perhaps drought selected for or against taxa with other traits besides those encoded by the functional genes we examined.

### Indirect effect: Litter origin

Litter origin also had no significant effect on frequencies of these fungal C- and N-targeting genes (Figs 3 and 4), even though drought had reduced the availability of cellulose and N in the leaf litter [3]. Accordingly, we rejected our third hypothesis that fungi with greater genetic capacity for cellulose and N acquisition should be favored on drought-derived litter, because they could better compete for potentially-limiting resources. Allison et al. [3] measured decomposition rates on these same samples, and reported no significant differences between drought-derived and control-derived litter. In addition, there was no significant effect of litter origin on fungal abundance (Fig 5).

In contrast, previous studies have reported that precipitation-induced changes in initial chemical composition of leaf litter can have consequences for decomposition rates. For example, in Hawaiian rainforests, trees in drier sites produced leaf litter with lower lignin and N concentrations than those in wetter sites [74, 75]. In turn, when all litter types decomposed in a common site, mass loss and N release progressed more quickly in the dry site-derived litter [74, 75]. In a global litter transplant experiment, initial litter chemistry was a better predictor of N release patterns during decomposition than was precipitation or actual evapotranspiration [76]. In the current study, perhaps drought-induced changes in litter chemical composition, although significant, were not large enough to influence microbial activity.

Other recent studies have examined relationships between climate and functional genes of bacteria, or bacteria + fungi combined [37, 77, 78]. In a rainfall exclusion experiment in Puerto Rico, drought increased the abundance of microbial genes related to degradation of chitin and cellulose [37]. In contrast, microbial genes encoding N and aromatic C metabolism declined with increasing aridity along a precipitation gradient in Israel [78]. Moreover, in a cross-biome sampling, microbial communities from deserts possessed fewer genes related to N and aromatic C metabolism than did those from non-deserts [77]. These studies collected standing, non-transplanted soil and microbes, so relationships may reflect a combination of direct environmental effects and indirect effects via changes in the microbial community or soil quality.

Belowground C and N cycling in ecosystems can be resilient to drought, even though water is a critical resource for soil microbes [10, 25–27, 33, 34, 79]. Our results suggest a possible underlying mechanism. In the short term, drought may select for fungi with the genetic capacity to break down and take up certain C and N compounds from litter. As a whole, fungi can be particularly drought-tolerant [1, 3, 9]. Indeed, they proliferated under dry conditions in this study. Even so, the shift toward higher genetic capacity for C and N cycling was more pronounced than the increase in fungal abundance under drought. Moreover, Berlemont et al. [8] noted that fungal genes became more prevalent in decomposing litter during the dry season in this field site. In this ecosystem, it is possible that not only were fungi drought-tolerant, but the drought-tolerant fungi were particularly capable of contributing to C and N cycling.

This study was conducted in a semi-arid ecosystem, and we recommend caution in extending these interpretations to other ecosystem types. Fungi in ecosystems commonly exposed to drought may be more likely to possess traits conferring tolerance to water stress [10, 80]. They may be more likely to respond positively to dry conditions than would fungi from wetter habitats. We also note that relatively short decomposition time in this experiment may have limited changes in DNA composition, and that RNA composition could have been more responsive in this time frame. Nevertheless, in a simultaneous companion experiment examining N fertilization in the same ecosystem, we observed changes in DNA composition in response to plot environment, microbe origin, and litter origin over this identical time frame [61]. Another issue to consider is that, to be conservative, we restricted our analyses to functional genes that were well-annotated in fungi/eukaryotes [40–45]. This narrow focus means that our analysis covers only a small fraction of reads from the sample. Thus, it is challenging to determine the ecological significance of increases in these small fractions. Currently, fewer than 1000 whole genomes of fungi have been published, and fungal gene annotation is nascent [39]. As fungal genomics progresses, we will hopefully be able to assess additional contributions of fungi to C and N dynamics.

### Conclusion

Altogether, the direct effect of drought in the plot environment on fungal functional genes consistently exceeded any indirect effects via shifts in the microbial community or changes in litter chemistry. Frequencies of amino acid permease, ammonium transporter, chitinase, and cellulose-targeting AA9 genes each increased significantly when common litter and microbes were incubated in drought plots versus control plots. Moreover, fungi from the phyla Ascomycota and Basidiomycota were alike in displaying increases in chitinase (Fig 3) and cellulose-targeting AA9 genes (Fig 4) with drought in the plot environment. These results tell a straightforward story: within a short time of exposure to drought, fungi with the genetic capacity for acquisition of these C- and N-compounds seemed to represent a larger portion of the microbial community in this California grassland. Fungal contributions to belowground C and N cycling may help maintain ecosystem functions such as litter decomposition during dry spells.

## Supporting information

S1 Table Statistical results for functional genes and fungal hyphal length. (DOCX)
