# Supplementary material for: Drought increases the frequencies of fungal functional genes related to carbon and nitrogen acquisition
Source: PLoS One. 2018 Nov 21;13(11):e0206441. doi: 10.1371/journal.pone.0206441 (PMC6248904; doi:10.1371/journal.pone.0206441)
Supplement: S1 Table — (DOCX) [file pone.0206441.s001.docx]

S1 Table. Statistical results for functional genes and fungal hyphal length.†

| Factor | F-ratio | P-value |  |  |
| --- | --- | --- | --- | --- |
| Ammonium transporter genes (# per 100,000 reads) | | | | |
| **Plot environment** | **F_1,8_ = 129** | **P < 0.001** |  |  |
| Litter origin | F_1,8_ = 0.363 | P = 0.564 |  |  |
| Microbe origin | F_1,8_ = 0.016 | P = 0.903 |  |  |
| Plot * litter | F_1,8_ = 0.268 | P = 0.618 |  |  |
| Plot * microbe | F_1,8_ = 0.086 | P = 0.776 |  |  |
| Litter * microbe | F_1,8_ = 0.143 | P = 0.715 |  |  |
| Plot * litter * microbe | F_1,8_ = 0.730 | P = 0.418 |  |  |
| Amino acid permease genes (# per 100,000 reads) | | | | |
| **Plot environment** | **F_1,8_ = 63.1** | **P < 0.001** |  |  |
| Litter origin | F_1,8_ = 0.411 | P = 0.539 |  |  |
| Microbe origin | F_1,8_ = 0.763 | P = 0.408 |  |  |
| Plot * litter | F_1,8_ = 0.263 | P = 0.622 |  |  |
| Plot * microbe | F_1,8_ = 0.008 | P = 0.930 |  |  |
| Litter * microbe | F_1,8_ = 0.318 | P = 0.588 |  |  |
| Plot * litter * microbe | F_1,8_ = 0.097 | P = 0.763 |  |  |
| Chitinase asco genes (# per 100,000 reads) | | | | |
| **Plot environment** | **F_1,8_ = 142** | **P < 0.001** |  |  |
| Litter origin | F_1,8_ = 0.678 | P = 0.434 |  |  |
| Microbe origin | F_1,8_ = 0.254 | P = 0.628 |  |  |
| Plot * litter | F_1,8_ = 0.090 | P = 0.772 |  |  |
| Plot * microbe | F_1,8_ = 0.227 | P = 0.646 |  |  |
| Litter * microbe | F_1,8_ = 0.335 | P = 0.579 |  |  |
| Plot * litter * microbe | F_1,8_ = 0.034 | P = 0.859 |  |  |
| Chitinase basidio genes (# per 100,000 reads) | | | | |
| **Plot environment** | **F_1,8_ = 65.6** | **P < 0.001** |  |  |
| Litter origin | F_1,8_ = 0.489 | P = 0.504 |  |  |
| Microbe origin | F_1,8_ = 0.520 | P = 0.491 |  |  |
| Plot * litter | F_1,8_ = 0.691 | P = 0.430 |  |  |
| Plot * microbe | F_1,8_ = 0.199 | P = 0.667 |  |  |
| Litter * microbe | F_1,8_ = 0.073 | P = 0.794 |  |  |
| Plot * litter * microbe | F_1,8_ = 0.056 | P = 0.819 |  |  |
| Cellulose-targeting AA9 asco genes (# per 100,000 reads) | | | | |
| **Plot environment** | **F_1,8_ = 131** | **P < 0.001** |  |  |
| Litter origin | F_1,8_ = 0.786 | P = 0.401 |  |  |
| Microbe origin | F_1,8_ = 0.331 | P = 0.581 |  |  |
| Plot * litter | F_1,8_ = 0.001 | P = 0.982 |  |  |
| Plot * microbe | F_1,8_ = 0.003 | P = 0.957 |  |  |
| Litter * microbe | F_1,8_ = 0.789 | P = 0.400 |  |  |
| Plot * litter * microbe | F_1,8_ = 0.070 | P = 0.798 |  |  |
| Cellulose-targeting AA9 basidio genes (# per 100,000 reads) | | | | |
| **Plot environment** | **F_1,8_ = 61.9** | **P < 0.001** |  |  |
| Litter origin | F_1,8_ = 0.005 | P = 0.944 |  |  |
| Microbe origin | F_1,8_ = 4.731 | P = 0.061 |  |  |
| Plot * litter | F_1,8_ = 0.757 | P = 0.410 |  |  |
| Plot * microbe | F_1,8_ = 0.757 | P = 0.410 |  |  |
| Litter * microbe | F_1,8_ = 1.431 | P = 0.266 |  |  |
| Plot * litter * microbe | F_1,8_ = 1.105 | P = 0.324 |  |  |
| Fungal hyphal length (m g^-1^) | | | | |
| **Plot environment** | **F_1,8_ = 5.196** | **P = 0.026** |  |  |
| Litter origin | F_1,8_ = 1.392 | P = 0.243 |  |  |
| **Microbe origin** | **F_1,8_ = 10.417** | **P = 0.002** |  |  |
| Plot * litter | F_1,8_ = 0.040 | P = 0.842 |  |  |
| Plot * microbe | F_1,8_ = 0.011 | P = 0.916 |  |  |
| Litter * microbe | F_1,8_ = 1.141 | P = 0.290 |  |  |
| **Plot * litter * microbe** | **F_1,8_ = 5.982** | **P = 0.018** |  |  |

†Significant effects are in bold.
